# A systematic genetic screen for genes involved in sensing inorganic phosphate availability in *Saccharomyces cerevisiae*

**DOI:** 10.1371/journal.pone.0176085

**Published:** 2017-05-17

**Authors:** Joonhyuk Choi, Abbhirami Rajagopal, Yi-Fan Xu, Joshua D. Rabinowitz, Erin K. O’Shea

**Affiliations:** 1 Faculty of Arts and Sciences Center for Systems Biology, Harvard University, Cambridge, Massachusetts, United States of America; 2 Department of Chemistry and Chemical Biology, Harvard University, Cambridge, Massachusetts, United States of America; 3 Howard Hughes Medical Institute, Harvard University, Cambridge, Massachusetts, United States of America; 4 Department of Molecular and Cellular Biology, Harvard University, Cambridge, Massachusetts, United States of America; 5 Department of Chemistry, Princeton University, Princeton, New Jersey, United States of America; 6 Lewis-Sigler Institute for Integrative Genomics, Princeton University, Princeton, New Jersey, United States of America; Texas A&M University College Station, UNITED STATES

## Abstract

*Saccharomyces cerevisiae* responds to changes in extracellular inorganic phosphate (P_i_) availability by regulating the activity of the phosphate-responsive (PHO) signaling pathway, enabling cells to maintain intracellular levels of the essential nutrient P_i_. P_i_-limitation induces upregulation of inositol heptakisphosphate (IP_7_) synthesized by the inositol hexakisphosphate kinase Vip1, triggering inhibition of the Pho80/Pho85 cyclin-cyclin dependent kinase (CDK) complex by the CDK inhibitor Pho81, which upregulates the PHO regulon through the CDK target and transcription factor Pho4. To identify genes that are involved in signaling upstream of the Pho80/Pho85/Pho81 complex and how they interact with each other to regulate the PHO pathway, we performed genome-wide screens with the synthetic genetic array method. We identified more than 300 mutants with defects in signaling upstream of the Pho80/Pho85/Pho81 complex, including *AAH1*, which encodes an adenine deaminase that negatively regulates the PHO pathway in a Vip1-dependent manner. Furthermore, we showed that even in the absence of *VIP1*, the PHO pathway can be activated under prolonged periods of P_i_ starvation, suggesting complexity in the mechanisms by which the PHO pathway is regulated.

## Introduction

In the face of dynamic and unpredictable fluctuations in nutrient availability, microorganisms achieve cellular nutrient homeostasis through the action of nutrient responsive signaling pathways [1]. P_i_ is an essential nutrient required for synthesis of ATP and cellular constituents such as phospholipids. *Saccharomyces cerevisiae* (budding yeast) responds to changes in extracellular P_i_ availability by regulating the activity of the PHO pathway. Cells repress the activity of the PHO pathway under high P_i_ conditions, whereas the PHO pathway is activated and induces expression of the PHO regulon under low P_i_ conditions, presumably to rectify a transient decrease in P_i_ concentration *in vivo* [2, 3]. For example, cells increase the rate of P_i_ uptake from the environment under low P_i_ conditions by upregulating expression of the acid phosphatase Pho5 [2, 4] and the high-affinity P_i_ transporter Pho84 [5].

**Fig 1:**
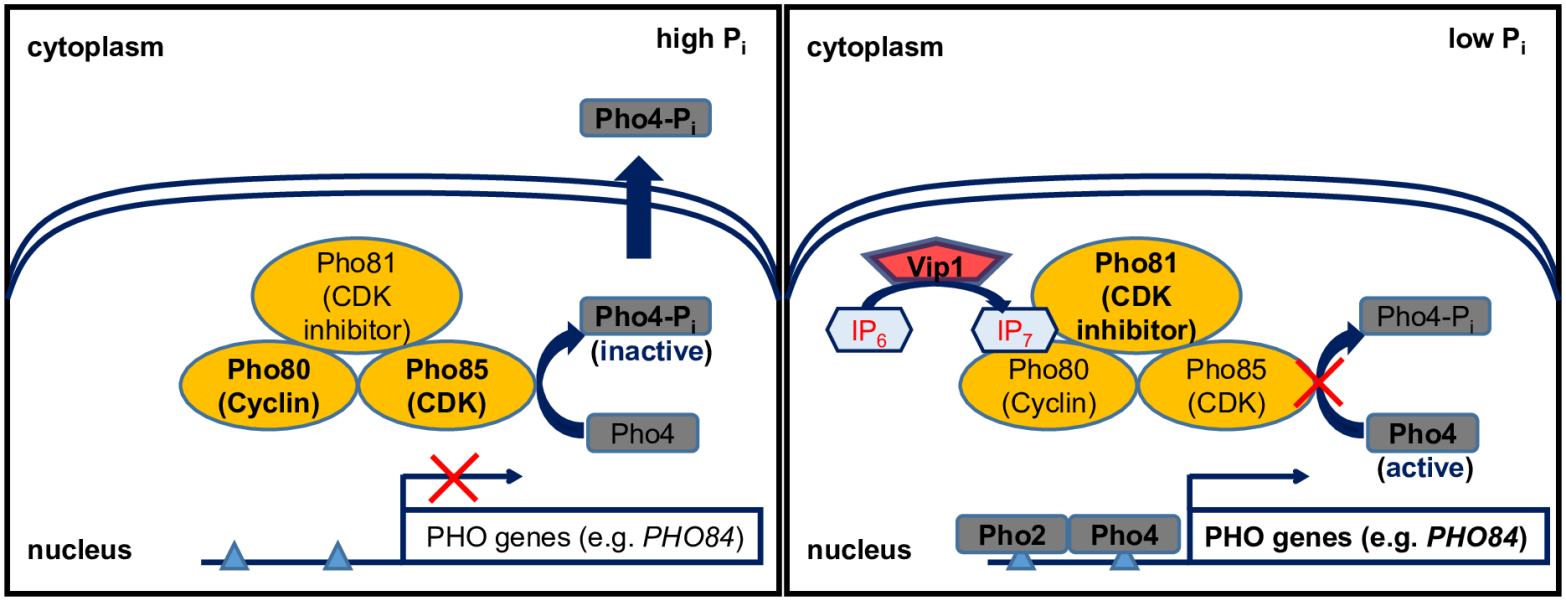
Transcriptional regulation of the PHO regulon in high and low P_i_ conditions.

The core regulatory complex of the PHO pathway consists of the cyclin Pho80, cyclin-dependent kinase (CDK) Pho85 and CDK inhibitor Pho81 [6–8] (Fig 1). Under high P_i_ conditions, the Pho81 inhibitor is not active and the Pho80/Pho85 complex phosphorylates the transcription factor Pho4, causing its export from the nucleus [9–12]. Under low P_i_ conditions, (1/3)–diphosphoinositol pentakisphosphate ((1/3)-PP-IP_5_; referred to as IP_7_) is produced by Vip1 and binds to Pho81, leading to inhibition of Pho80/Pho85 complex kinase activity [13, 14], dephosphorylation and nuclear localization of Pho4, and transcriptional activation of the PHO regulon, including *PHO5* and *PHO84* [15].

Compared to the well-elucidated pathway downstream of the Pho80/Pho85/Pho81 complex, little is known about upstream signaling processes. We do not understand how P_i_ availability is sensed and how information about P_i_ availability is transmitted to enzymes that regulate IP_7_ levels. Only three genes have been implicated in upstream signaling: genes encoding the adenosine kinase Ado1, the adenylate kinase Adk1, and the PP-IP_5_ kinase Vip1 [14, 16]. However, we do not understand how these enzymes are regulated under different P_i_ conditions or how they interact with each other to regulate the PHO pathway. Furthermore, other players beyond these three enzymes remain unknown.

To identify genes involved in signaling process upstream of the Pho80/Pho85/Pho81 complex, a previous study performed a high-throughput and quantitative screen of the yeast deletion library, consisting of 4848 haploid strains deleted for non-essential genes, searching for novel mutants defective in *PHO5* expression [16]. Of the 90 most statistically significant candidates identified in the screen, 19 mutants were defective in *PHO5* expression in a PHO-pathway specific manner, with *ado1Δ* and *adk1Δ* being the only mutants defective in signaling processes upstream of the Pho80/Pho85/Pho81 complex. *VIP1*, another upstream signaling factor [14], was not identified, suggesting that the screen was not comprehensive and more genes are left to be identified.

To perform genome-wide genetic screening of the PHO pathway, we adopted the synthetic genetic array (SGA) method. Originally, this methodology was developed for systematic construction of double mutants to create a global genetic interaction map [17–20]. The SGA method allows us to query large numbers of mutants. Additionally, by introducing a fluorescent reporter into each mutant in the library, we can improve the sensitivity and quantitative nature of the PHO pathway readout. Finally, we can investigate functional relationships between two genes from double mutant analysis, which compares the phenotype of a double mutant with that of its single mutants and estimates the extent to which deletion of one gene affects the phenotype of another deletion [17, 20].

Employing the SGA method, we identified more than 300 mutants defective in upstream signaling of the PHO pathway. In particular, we found that deletion of *AAH1*, whose product is involved in adenine nucleotide metabolism, de-represses the PHO pathway under high P_i_ conditions. We also characterized functional relationships between mutants such as *aah1Δ* and others defective in upstream signaling of the PHO pathway and showed that *aah1Δ* requires Vip1 for constitutive activation of the PHO pathway.

## Materials and methods

### Strains

All strains for screening used in this study are in the BY4741 background. A yeast library was obtained from the Weissman lab at UCSF consisting of 4974 knockout alleles of non-essential genes and 878 hypomorphic alleles of essential genes [17, 21]. All strains in the library are MATa haploids.

The *PHO84* reporter strain was generated from yMJ003 (MATα *his3Δ1 leu2Δ0 met15Δ0 ura3Δ0 LYS+ can1Δ*::*STE2*pr-*spHIS5 lyp1Δ*::*STE3*pr-*LEU2 cyh2 ura3Δ*::UPRE-GFP-*TEF2*pr-RFP-*MET15-URA3*) [17]. *UPRE-GFP* sequence in yMJ003 was replaced with *PHO84* promoter sequence taken from -1000 to -1 base pairs from the ATG of the *PHO84* open reading frame followed by Venus fluorescence protein sequences from pKT0090 [22]. To reduce *PHO80* mRNA stability, the DAmP (Decreased Abundance by mRNA Perturbation) *pho80* strain, *pho80DΔ* [23], was generated by inserting a nourseothricin marker (Nat^R^) obtained from pFA6a-natMX4 [24] right after the stop codon of *PHO80*.

### Insertion of the *PHO84* reporter into each strain in the yeast library and generation of double mutants

The SGA method [17, 19] was applied to introduce the *PHO84* reporter into each strain in the yeast library; the protocol for this process was the same as described in [17]. The *PHO84* reporter strain was crossed to each of 5,852 strains in the library harboring G418 marker (Kan^R^) in parallel with replicate-pinning tools (V&P Scientific, INC). After crossing, diploids carrying both the *PHO84* reporter and the mutations (knockouts or hypomorphic alleles) were selected on SD—ura + G418 plates and put on sporulation plates. After sporulation, final MATα haploids carrying both the *PHO84* reporter and Kan^R^ marker for the mutations were selected on SD—ura + G418 –leu—arg—lys + canavanine (Sigma-aldrich) + S-(2-Aminoethyl)-L-cysteine (Sigma-aldrich) + mono sodium glutamate (Sigma-aldrich) plates.

Double mutants were generated by crossing chosen MATα haploids carrying both the *PHO84* reporter and Kan^R^ marker to other MATa haploids of interest whose knockout marker was Nat^R^ [24]. The selection process was identical to the case where the *PHO84* reporter was inserted into the yeast library except that both nourseothricin (ClonNAT, Werner BioAgents) and G418 were used to simultaneously select for the Nat^R^ and Kan^R^ markers.

### *PHO84* expression level measurements from mutants

Cells were grown in synthetic complete (SC) medium at 30°C in a 384 well-plate on a plate shaker. SC medium with different P_i_ concentrations was prepared as described in [25]. Strains were inoculated from final haploid selection plates into 80 μl of 10 mM P_i_ SC medium and were grown overnight. To reduce P_i_ spillover from the overnight cultures, 5 μl of overnight cell culture was first inoculated into 75 μl of no P_i_ medium using a BioMek FX liquid handling robot (Beckman Coulter, Inc.,Fullerton, CA, USA). Subsequently, 5 μl of the inoculated cultures in no P_i_ medium were inoculated again into 75 μl of 50 μM and 1 mM P_i_ medium and were grown for 8 hours to measure the *PHO84* expression levels of each strain. 50 μM P_i_ is near the maximum P_i_ concentration that leads to activation of the PHO pathway and 1 mM P_i_ is near the minimum P_i_ concentration to repress the PHO pathway. Note that the bimodality in *PHO84* expression in the wild type strain arises in the conditions ~ 150 to 250 μM P_i_ (intermediate P_i_); bimodality results in an off-population that expresses little *PHO84* and an on-population that highly expresses *PHO84* [26]. The intermediate P_i_ concentration regime was avoided because the relative ratio of the off- to on-population was too sensitive to small changes in P_i_ concentration to get reliable readouts from the measurements.

The *PHO84* expression level of each strain was measured three times with a flow cytometer. The cell cultures were transferred to a Becton Dickinson High Throughput Sampler (BD, Franklin Lakes, NJ USA), which directly injected cells from the wells of the plate into the LSR Fortessa flow cytometer (BD). It took about 110 minutes to measure one 384-well plate and 2,000–6,000 cells/well were measured. Samples with low cell counts (<250/well) were disregarded. Venus was excited at 488nm and its fluorescence was collected through a 505 nm long-pass filter and a HQ 515/20 band-pass filter (YFP channel). mCherry was excited at 532 nm and fluorescence was collected through a 600nm long-pass filter and a 610/20 band-pass filter (RFP channel).

### Extraction of the mean *PHO84* reporter level of each strain from flow cytometry data

A customized MATLAB code was written to calculate the mean *PHO84* reporter level of each strain from flow cytometry data. To import raw “.fcs” files obtained from the flow cytometer to our customized code, a function to read FCS 3.0 format written by Laszlo Balkay was used (available at the community File Exchange section at http://www.mathworks.com/matlabcentral). To adjust for non-P_i_-specific perturbation of single-cell *PHO84* expression levels (e.g. due to the different cell-cycle stages of cells), the signal in the YFP channel (*PHO84* signal) was normalized by the signal in the RFP channel (*TEF2* signal) for every cell. The distribution of log_2_(YFP/RFP) was obtained over all cells in the population and 5% of cells at either end of the distribution were removed to eliminate outliers. Then, the log_2_(YFP/RFP) of the remaining 90% of the cells was averaged to represent the *PHO84* reporter level of each strain. Note that the mean value, not the median, of the cell population was calculated for the *PHO84* reporter level of the sample. The distributions of the *PHO84* signals in the YFP channel of some mutants were bimodal such that the median of those mutants was very sensitive to the relative ratio of the on- and off-populations and could differ greatly even if the ratio changed slightly due to measurement noise. All of the extracted mean *PHO84* reporter levels measured in this study were reported in S1 Table.

### Normalization of the mean *PHO84* reporter levels of each strain

The mean *PHO84* reporter level of each strain extracted from flow cytometry was normalized as described in [17]. In the genome-wide single mutant screen in 50 μM and 1mM P_i_ conditions, the mean *PHO84* reporter levels of the single mutants were normalized by the median of the mean *PHO84* reporter levels of all samples on the same plate. This normalization process is based on the premise that the number of genes expected to be involved in regulation of the PHO pathway is much smaller than the number of the strains in the library. In the epistasis analysis with *pho80DΔ* and *pho81Δ*, the mean *PHO84* reporter levels of each double mutant were normalized by the value of *pho80DΔ* and *pho81Δ*, respectively. For the double mutant analysis with *ado1Δ* and *aah1Δ*, the wild type was included in 6 wells in the plate. The mean *PHO84* reporter levels of the 6 wild type samples were averaged and the resulting value was used to normalize the mean *PHO84* reporter levels of the double mutants.

### Calculation of p-values for measurement errors

P-values for measurement errors in the genome-wide screen were calculated as described in [17]. The distribution of measurement errors was defined as (1-c1)*norm(0,σ_1_) + c1*norm(0,σ_2_) where c1 is the coefficient and norm(0,σ) is a Gaussian distribution with standard deviation σ and mean zero. C1, σ_1_, and σ_2_ were obtained using an iterative nonlinear fit to actual distribution of the difference between three replicate measurements of reporter levels in the library. The calculated distribution of measurement errors was used to generate the expected distribution of measured values for a strain with wild type *PHO84* reporter levels [17]. Using this distribution, a p-value was calculated as a function of a measured reporter level (L) and number of measurements (N), an estimate of the probability of observing a reporter level equal to or more extreme than L upon averaging of N independent measurements of the wild type strain [17]. Strains with p-values <10^−3^ were designated as those showing *PHO84* expression levels different from the wild type in 50 μM or 1mM P_i_ conditions.

### Extraction of ATP, ADP, and AMP

The concentrations of ATP, ADP, and AMP were measured as described in [27]. Overnight cultures with O.D. 600 < 0.3 were diluted into 100 ml of fresh 10 mM P_i_ liquid medium and grown for at least 12 hours before harvest. The final O.D. 600 was ~ 0.2. 20 ml of this cell culture was used to extract metabolites from the wild type, *adk1****Δ***, *aah1****Δ***, and *ado1****Δ*** mutants grown in 10 mM P_i_. For the time course measurements of the wild type in no P_i_, the rest of the cell culture was transferred onto a vacuum filtration apparatus (Millipore nitrocellulose membrane with 0.8 μm pore size, Cat No. AAWG025000), washed with 30 ml of no P_i_ medium and resuspended into fresh no P_i_ medium. The resuspended cell culture was inoculated into fresh 20 mL of no P_i_ medium such that the final O.D. 600 at each time point (5, 15, 30, 60, and 90 minutes) was 0.2. For harvesting, cell culture was filtered onto a 50 mm nylon membrane filter, which was immediately transferred into -20°C extraction solvent (40:40:20 acetonitrile/methanol/water).

Cell extracts were analyzed by reversed phase ion-pairing liquid chromatography (LC) coupled with electrospray ionization (ESI) (negative mode) in a high-resolution, high-accuracy mass spectrometer (MS) (Exactive; Thermo Fisher Scientific) [28]. It was operated in full scan mode at 1 s scan time, 10^5^ resolution, with compound identities verified by mass and retention time match to authenticated standard [28].

### Calculation of the relative concentrations of ATP, ADP, and AMP

To convert raw LC-MS/MS ion counts to relative cellular concentrations, ion counts were first normalized by the cell density [29]. The normalized ion counts were converted to relative concentration by dividing the value for the samples by the corresponding value of the reference data (S2 Table) [29]. For the time course measurements in no P_i_, data for each time point were divided by the corresponding values at time 0. For the measurements of *adk1****Δ***, *aah1****Δ***, and *ado1****Δ*** mutants, the values of the mutants were divided by the values of the wild type strain grown in 10 mM P_i_.

### Determination of array strains for double mutant analysis with *ado1Δ* and *aah1Δ*

320 array strains (293 less induced hits and 27 less repressed hits) exhibiting at least a 2-fold change in *PHO84* expression levels compared to the wild type were selected for double mutant analysis. In addition, other mutants functionally related to one of the 320 array strains were included in the analysis, even though their *PHO84* expression levels did not satisfy the conditions to be selected for the genome-wide single mutant screen. For example, *ipk1Δ* was included as an array strain as Ipk1 produces IP6 –a precursor for IP_7_ [30]–even though the *PHO84* expression level of *ipk1Δ* is similar to that of wild type in 50 μM P_i_ (S3 Table). All strains measured as double mutants with *ado1Δ* and *aah1Δ* were listed in S4 Table.

## Results

### Design of screen to identify genes acting upstream of the Pho80/Pho85/Pho81 complex

We carried out two steps of systematic screening to identify genes involved in upstream PHO pathway signaling. First, we identified mutants defective in regulation of *PHO84* expression in a yeast library containing deletion mutants of non-essential genes and hypomorphic alleles of essential genes. We chose *PHO84* expression as a reporter instead of *PHO5* because it is a more sensitive readout of pathway activity; since *PHO5* expression requires more severe P_i_-limited conditions than does *PHO84* expression [25], mutants defective in *PHO5* expression are a subset of those defective in *PHO84* expression. We looked for mutants expressing less *PHO84* than the wild type in low P_i_ (less induced hit) and mutants expressing more *PHO84* than the wild type in high P_i_ conditions (less repressed hit). Second, to identify the subset of mutants defective in signaling upstream of the Pho80/Pho85/Pho81 complex, we performed epistasis analysis, which can determine the order of action between genes.

### Genome-wide single mutant screening for mutants with altered *PHO84* expression

**Fig 2:**
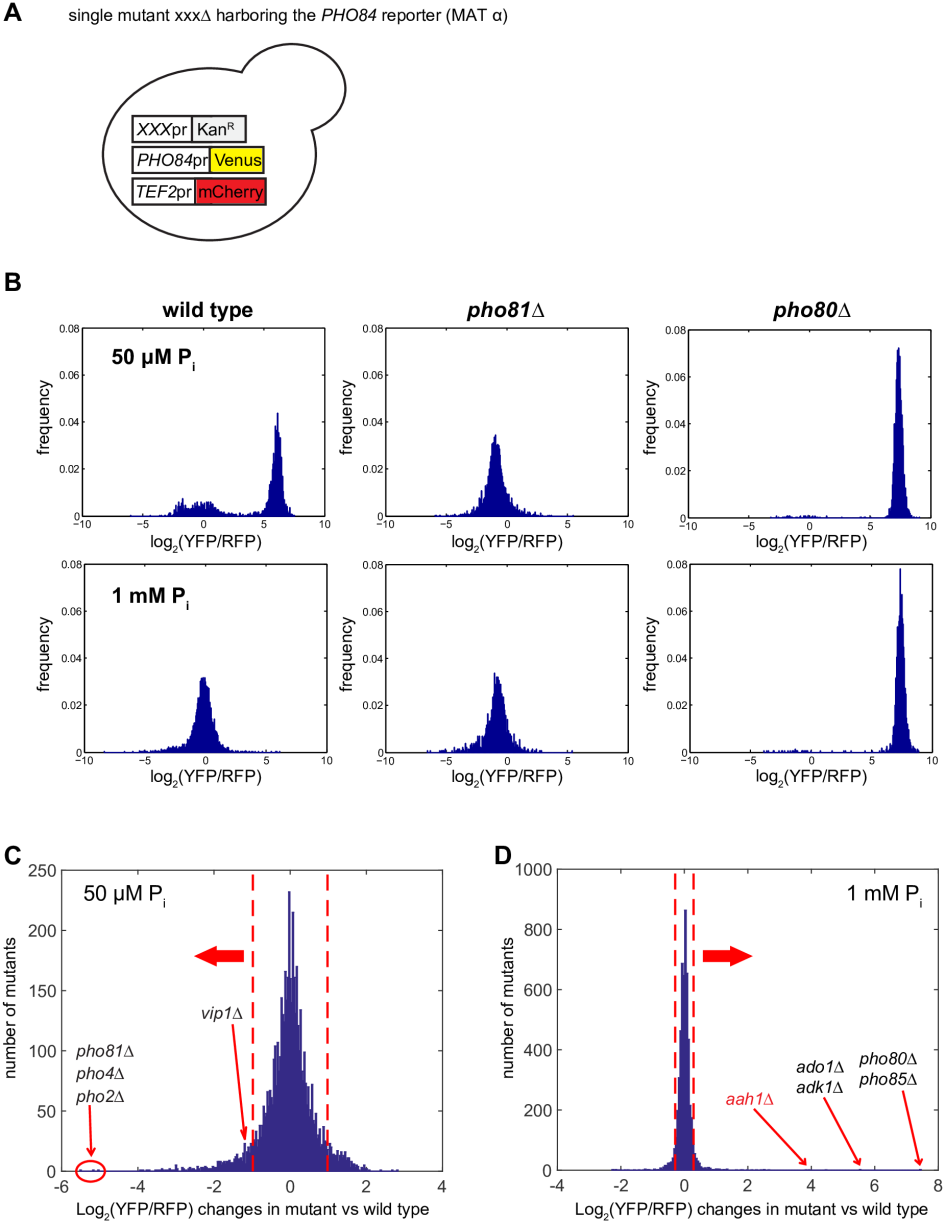
Identification of mutants with altered *PHO84* expression in low and high P_i_ conditions. (A) Generation of single mutants harboring the *PHO84* reporter with the SGA method. The *PHO84* reporter consists of *PHO84* promoter-driven Venus and *TEF2* promoter-driven mCherry. Each single mutant in the library denoted by *xxx*Δ is kanamycin (G418)-resistant. (B) The distributions of the *PHO84* reporter levels in single cells in the *pho81Δ* and *pho80Δ* strains. Log_2_ intensity ratio of Venus to mCherry (log_2_(YFP/RFP)) was used to quantify the *PHO84* expression level. (C, D) The *PHO84* reporter levels of single mutants in the library measured in 50 μM P_i_ and 1 mM P_i_ conditions. The *PHO84* reporter level of each mutant was normalized to that of the wild type value in each P_i_ concentration (Materials and methods). Red dashed lines in (C) and (D) indicate the *PHO84* reporter levels with p-values less than 0.001 estimating the maximum range of the *PHO84* reporter levels that the wild type exhibits in each P_i_ concentration. The mutants in black are previously identified mutants and the one in red is identified in this study.

To measure the *PHO84* expression level of each mutant in the library, we first constructed a reporter in which the *PHO84* promoter drives expression of the yellow fluorescence protein (YFP) Venus (Fig 2A). To correct for P_i_-independent expression changes, we co-expressed the red fluorescence protein (RFP) mCherry driven by the *TEF2* constitutive promoter (Materials and methods), and used the log_2_ intensity ratio of YFP/RFP as a proxy for the activity of the PHO pathway (hereafter referred to as the *PHO84* reporter level). We inserted the reporters into each mutant in the library using the SGA method (Fig 2A and Materials and methods) [17], obtained haploid strains harboring the reporters and each mutation, and then measured fluorescence from those haploid strains using flow cytometry (Materials and methods). To identify mutants with subtle defects such as different P_i_ activation/repression thresholds, as well as mutants with severe defects such as complete repression or constitutive derepression of the PHO pathway, we screened cells grown in 50 μM and 1 mM P_i_, which are near the P_i_ threshold concentrations to turn on and off the PHO pathway, respectively. We validated our screening design by verifying that we could reproduce the phenotypes of mutants known to impair the activity of the PHO pathway such as *pho80Δ* and *pho81Δ* (Fig 2B–2D). Of the 19 mutants identified previously [16], 12 were detected with a reliable number of cells at least twice in this study and the PHO phenotypes of all 12 mutants were recapitulated.

Calculating p-values of measurement errors, we estimated the maximum range of the *PHO84* reporter levels (-0.98 and 0.29; red dashed lines in Fig 2C and 2D) that the wild type exhibits in low P_i_ (50 μM) and high P_i_ (1 mM) (Materials and methods) and used these as thresholds to identify mutants (“hits”) expressing *PHO84* at levels different from those of the wild type. We identified 380 less induced hits from low P_i_ which exhibited the *PHO84* reporter levels less than -0.98, and 243 less repressed hits from high P_i_ which exhibited the *PHO84* reporter levels more than 0.29 (S3 Table).

### Epistasis analysis to identify genes acting upstream of *PHO80* or *PHO81*

To identify genes acting upstream of the Pho80/Pho85/Pho81 kinase complex from the 623 hits (380 less induced and 243 less repressed), we performed epistasis analysis. Epistasis refers to a genetic interaction in which mutation of one gene influences the phenotypic effects of another [20, 31]; this approach can be used to infer the order of gene action in a signaling pathway [17]. For example, we can learn that gene A acts upstream of gene B from the observation that deletion of gene B masks the phenotypic effect of deletion of gene A.

**Fig 3:**
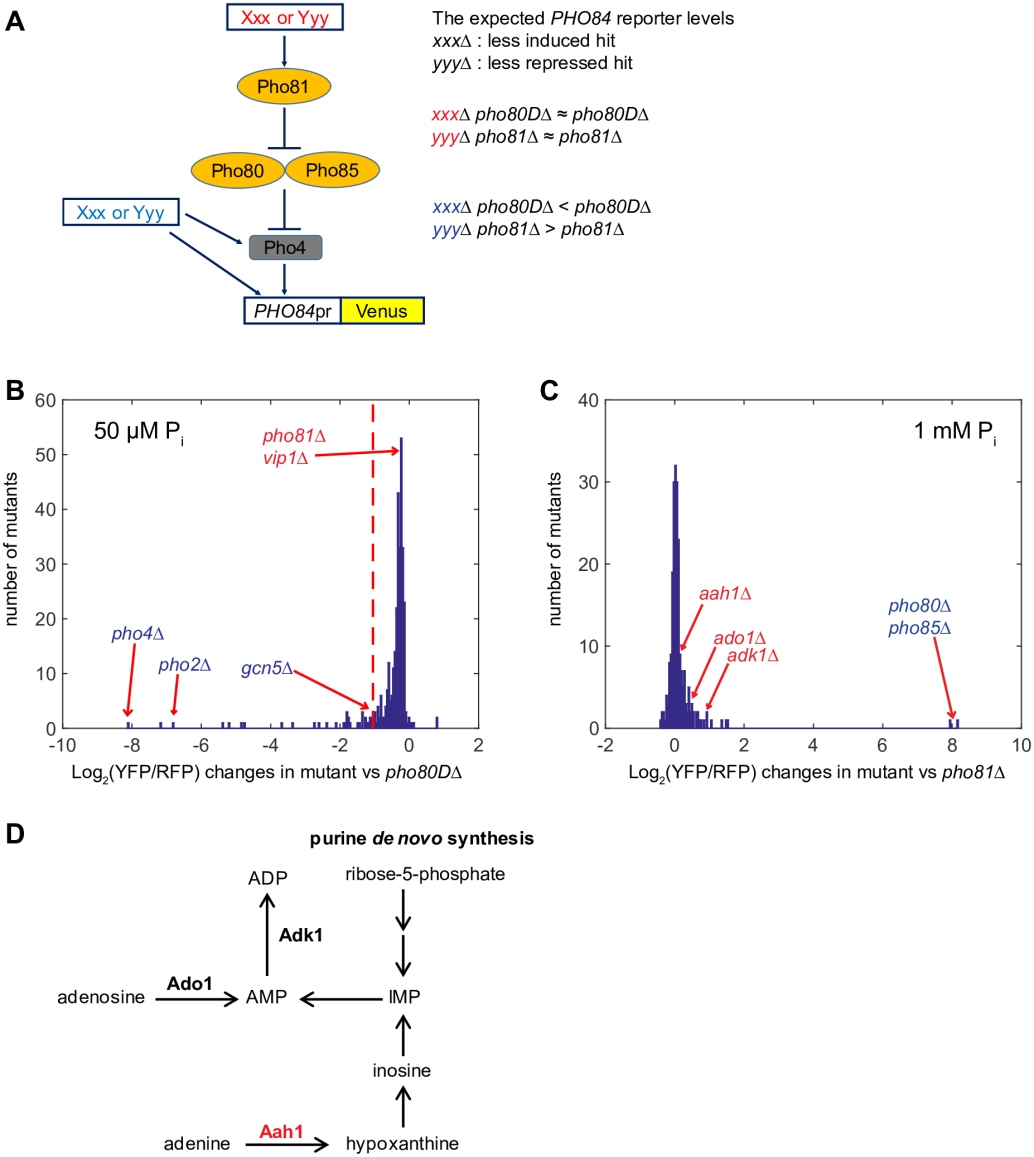
Identification of genes acting upstream of the Pho80/Pho85/Pho81 kinase complex. (A) A schematic diagram depicting the expected outcome of epistasis analysis depending on whether or not a mutant is defective in the signaling process upstream of Pho80/Pho85. Genes in red act upstream of Pho80/Pho85 and those in blue do not act upstream of Pho80/Pho85. (B) The *PHO84* reporter levels of double mutants carrying the less induced hits and *pho80DΔ* in 50 μM P_i_ conditions. All 380 less induced hits were used to generate the double mutants. The *PHO84* reporter levels of double mutants in (B) were normalized to that of *pho80DΔ*. A red dashed line in (B) indicates the maximum *PHO84* reporter level of double mutants generated by one of the known downstream genes (*pho80DΔ gcn5Δ*). (C) The *PHO84* reporter levels of double mutants carrying the less repressed hits and *pho81Δ* in 1 mM P_i_ conditions. All 243 less repressed hits were used to generate the double mutants. The *PHO84* reporter levels of double mutants in (C) were normalized to that of *pho81Δ*. In (B) and (C), mutants in blue and red are defective in signaling process downstream and upstream of Pho80/Pho85, respectively. (D) A schematic diagram depicting adenine nucleotide metabolism. A gene in red is identified in this study and those in bold black are previously identified.

For the less induced hits, we performed epistasis analysis in low P_i_ by generating double mutants with *pho80*. If the less induced gene hit *XXX* acts upstream of *PHO80*, mutation of *PHO80* masks the effect of reduced *PHO84* expression such that the *xxxΔ pho80* double mutant expresses *PHO84* at a level similar to the *pho80* single mutant (Fig 3A). For this analysis we used *pho80DΔ*, a strain with attenuated *PHO80* mRNA expression (Materials and methods) instead of complete deletion (*pho80Δ*) since *PHO84* expression in the *pho80Δ* strain is extremely strong (Fig 2B and 2D). We hypothesized that *pho80DΔ* would sensitize *PHO84* expression to the PHO pathway mutants so that epistasis analysis with *pho80DΔ* would allow us to identify genes acting upstream of *PHO80*.

We validated our strategy for epistasis analysis with *pho80DΔ* by comparing the *PHO84* reporter level of *pho80DΔ* with those of double mutants carrying mutations in genes known to act downstream or upstream (Fig 3B). Consistent with our hypothesis, *pho80DΔ pho81Δ* and *pho80DΔ vip1Δ*, which carry deletion mutations of upstream genes, expressed *PHO84* at a level similar to *pho80DΔ* (-0.19 and -0.19, respectively; S5 Table). By contrast, double mutants of downstream genes expressed less *PHO84* than did *pho80DΔ* (Fig 3B). For example, *pho80DΔ pho4Δ* expressed the lowest level of *PHO84* (-8.13, S5 Table) of all the double mutants and *pho80DΔ gcn5Δ* (a catalytic subunit of ADA and SAGA histone acetyltransferase complexes [32, 33]) expressed *PHO84* at levels lower than *pho80DΔ* (-1.07, S5 Table). To identify upstream genes, we needed to determine the lowest *PHO84* reporter level, as a threshold, that double mutants of *pho80DΔ* and true upstream genes could exhibit. This threshold should lie between -1.07 and -0.19 since -1.07 is the maximum *PHO84* reporter level of double mutants of *pho80DΔ* and known downstream genes (*pho80DΔ gcn5Δ*) and -0.19 is the *PHO84* reporter level of double mutants of *pho80DΔ* and known upstream genes such as *pho80DΔ vip1Δ*. To try and ensure that no hits were lost due to a stringent threshold, we used -1.07 as the threshold in spite of the fact that this approach undoubtedly will allow false-positives. We classified 300 of the measured 334 less induced hits expressing *PHO84* above the threshold as defective in signaling upstream of *PHO80* (S5 Table).

For the less repressed hits, we performed epistasis analysis in high P_i_ with *pho81Δ* (Fig 3C). If gene *YYY* acts upstream of *PHO81*, deletion of *PHO81* masks increased *PHO84* expression such that *pho81Δ yyyΔ* expresses *PHO84* at a level similar to *pho81Δ* (Fig 3A). As shown in Fig 3C, *pho81Δ adk1Δ* and *pho81Δ ado1Δ*, which carry deletions of genes known to act upstream of *PHO81*, expressed *PHO84* at a level similar to *pho81Δ* (0.92 and 0.48, respectively; S6 Table). By contrast, *pho81Δ pho80Δ* and *pho81Δ pho85Δ*, which carry deletion mutation of genes known to act downstream of *PHO81*, expressed *PHO84* at higher levels than *pho81Δ* (7.95 and 8.18, respectively; S6 Table). Because *pho81Δ pho80Δ* and *pho81Δ pho85Δ* were the only double mutants expressing *PHO84* at significantly higher levels than *pho81Δ*, we classified the remaining 222 of the measured 224 less repressed hits as defective in signaling upstream of *PHO81* (S6 Table).

### Deletion of *AAH1* encoding an adenine deaminase involved in adenine nucleotide metabolism derepresses the PHO pathway in high P_i_

One of the genes we identified with the strongest phenotype was *AAH1*, a gene encoding an adenine deaminase that converts adenine into hypoxanthine. *AAH1* acts upstream of *PHO80* and loss of *AAH1* leads to >15-fold induction of *PHO84* expression in high P_i_ (Figs 2D and 3C, S1A Fig, S6 Table). Together with *ADO1* and *ADK1*, *AAH1* is involved in adenine nucleotide metabolism (Fig 3D). Based on the observation that three mutants defective in adenine nucleotide metabolism share the same PHO phenotype, it is plausible that intermediates or products of adenine nucleotide metabolism act as signaling factors for the PHO pathway.

**Fig 4:**
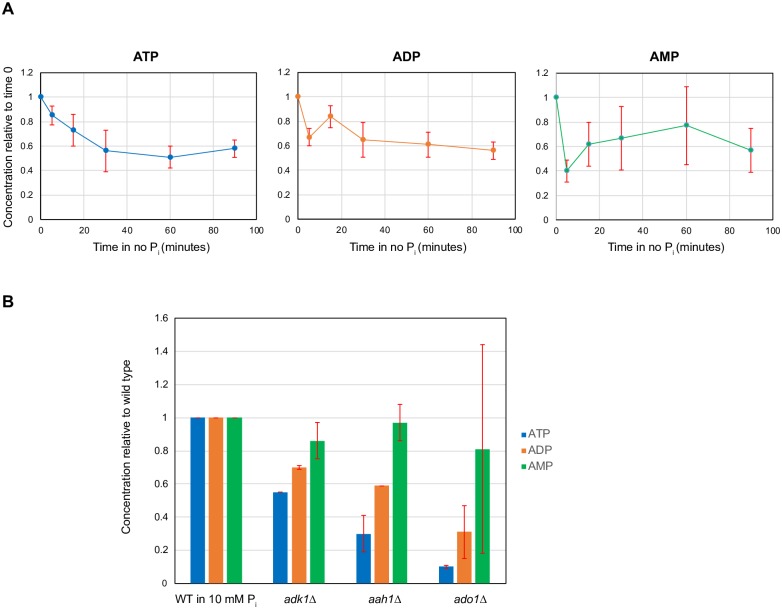
Adenine nucleotide levels in the wild type in no P_i_ and in *adk1Δ*, *aah1Δ* and *ado1Δ* in high P_i_. (A) [ATP], [ADP], and [AMP] in wild type (WT) cells over time grown in no P_i_ medium. All the adenine nucleotide concentrations at each time point were normalized to those in 10 mM P_i_. Note that the PHO pathway in no P_i_ is activated within 15 minutes. Adenine nucleotide levels at each time point were measured three times and the error bars in (A) are standard errors. (B) [ATP], [ADP], and [AMP] in WT, *adk1Δ*, *aah1Δ*, and *ado1Δ* in 10 mM P_i_. Adenine nucleotide levels in the three mutants were measured two times and the error bars in (B) are standard errors.

Based on a previous metabolome study in yeast [29], we speculated that a low ATP level might be involved in activation of the PHO pathway. Measuring the metabolic profiles in steady-state, chemostat grown cultures of *S*. *cerevisiae* with 3 different limiting nutrients (carbon, nitrogen and P_i_), Boer et al. showed that metabolite concentrations were highly sensitive to the identity of the limiting nutrient, with P_i_ limitation leading to low nucleotide levels [29]. Furthermore, particularly strong responses occurred in metabolites closely linked to the limiting nutrient, for example, ATP in P_i_ limitation [29]. To test if we observe metabolic changes in response to changes in P_i_ availability in non-steady state experiments, we measured [ATP], [ADP], and [AMP] over time after transferring wild type cells from high P_i_ to no P_i_ medium (Materials and methods). As controls where the PHO pathway is activated, we also measured the adenine nucleotide levels in *adk1Δ*, *aah1Δ*, and *ado1Δ* grown in high P_i_ and compared them with those of the wild type grown in no P_i_ (Materials and methods). Consistent with the chemostat measurements [29], the adenine nucleotide levels of cells grown in no P_i_ were lower than those of cells grown in high P_i_ and ATP levels monotonically decreased over time in cells grown in no P_i_ (Fig 4A, S2 Table). Furthermore, ATP levels in *adk1Δ*, *aah1Δ*, and *ado1Δ* grown in high P_i_ were comparable to those in the wild type grown in no P_i_ (Fig 4B, S2 Table). The timescale of the reduction in the ATP level parallels the timescale for activation and nuclear accumulation of Pho4: after 15 minutes in no P_i_ ATP levels were reduced by ~25% relative to levels in 10 mM P_i_ and 50% of cells exhibited Pho4 nuclear localization, whereas after 60 minutes ATP levels were reduced by ~50% and essentially all cells had Pho4 localized to the nucleus [34]. These measurements support the speculation that a low ATP level might be involved in activation of the PHO pathway under low P_i_ conditions.

### The PHO pathway in *vip1Δ* can be activated in a long period of time under P_i_-limited conditions

**Fig 5:**
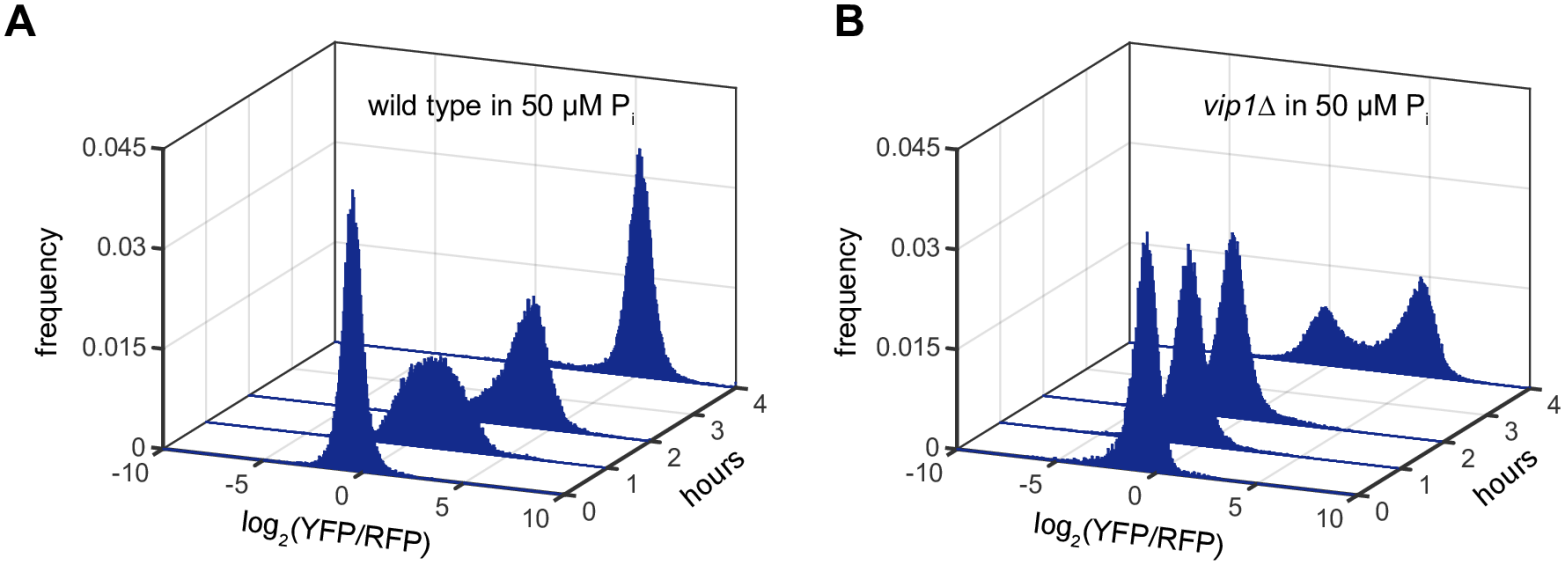
The PHO pathway in *vip1Δ* mutant is inducible in 50 uM P_i_, but its induction kinetics are slower than the wild type. (A) The *PHO84* reporter levels of the wild type over time in 50 uM P_i_. (B) The *PHO84* reporter levels of *vip1Δ* over time in 50 uM P_i_. Time 0 data in (A) and (B) were obtained in 10 mM P_i_ before cells were inoculated into 50 μM P_i_.

In this study, we observed that *vip1Δ* was able to induce *PHO84* expression after 8 hours in low P_i_, although to a lesser extent (~ 55%) than the wild type (Fig 2C, S1B Fig). This observation seems inconsistent with a previous study showing that deletion of *VIP1* prevents activation of the PHO pathway under low P_i_ conditions [14]. However, the earlier work assayed pathway activity at 2 hours, rather than 8 hours, suggesting that *PHO84* induction kinetics in *vip1Δ* are slower than in the wild type. To test if this is true, we measured *PHO84* expression levels in *vip1Δ* in low P_i_ medium over time and compared them with those in the wild type strain. As shown in Fig 5, the wild type strain expressed *PHO84* after 1 hour in low P_i_ medium but *vip1Δ* started to induce *PHO84* expression only after 4 hours. Therefore, we conclude that *vip1Δ* is inducible, but activation of the PHO pathway in *vip1Δ* is slower than in the wild type.

### *VIP1* is required for constitutive activation of the PHO pathway in both *ado1Δ* and *aah1Δ* mutants

**Fig 6:**
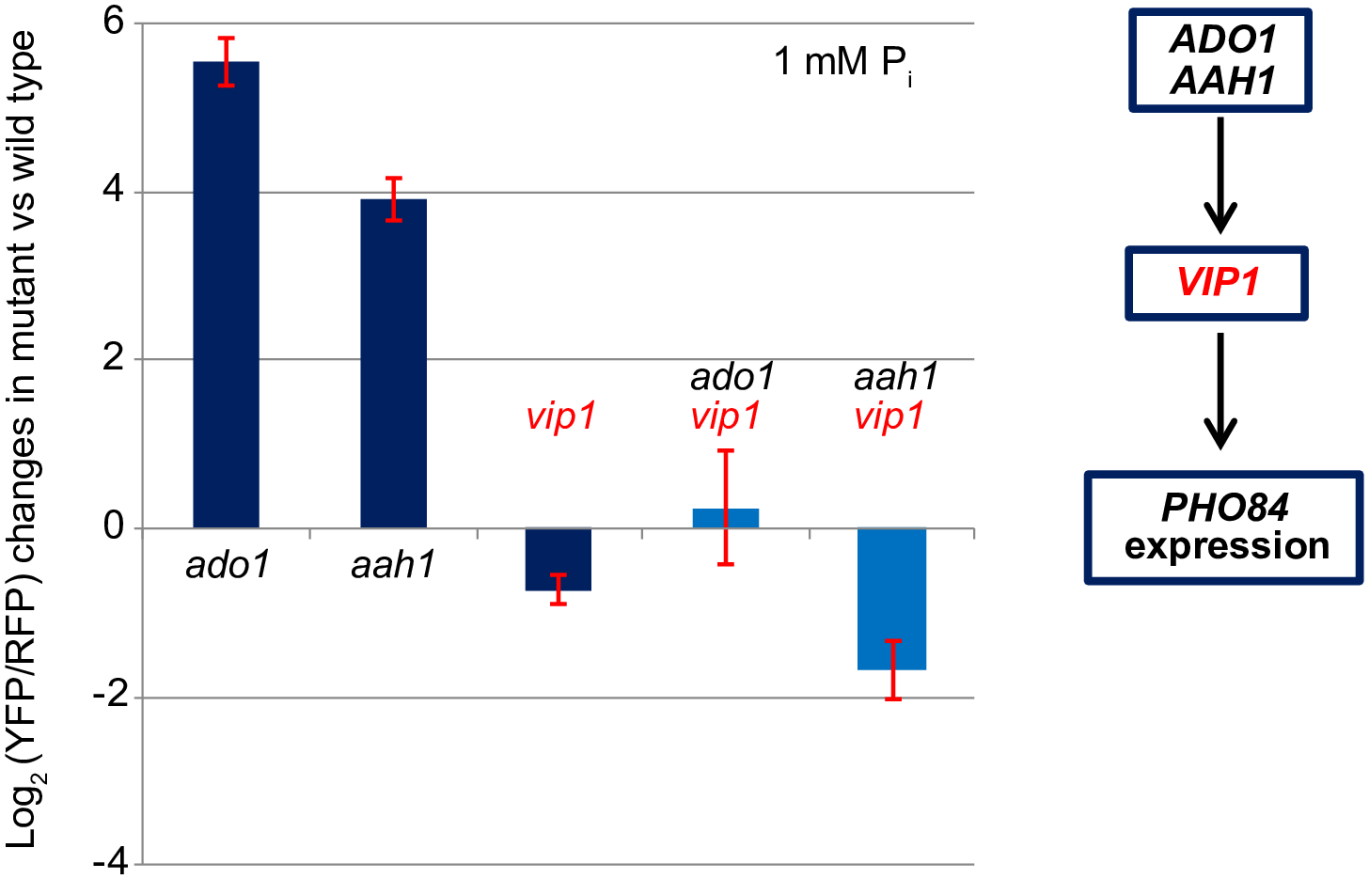
*VIP1* is required for constitutive activation of the PHO pathway in *ado1Δ* and *aah1Δ*. The *PHO84* reporter levels in all the strains in Fig 6 were averaged over 3 measurements and normalized to the *PHO84* reporter level in the wild type in 1 mM P_i_ conditions. Error bars represent the standard deviation of the normalized *PHO84* reporter levels in the mutants.

To understand the molecular mechanisms underlying the PHO phenotypes of *ado1Δ*, *aah1Δ*, and *adk1Δ* mutants defective in adenine nucleotide metabolism (Fig 3D), we sought to identify genes required for the constitutive activation phenotype of the three mutants. First, we tried to generate double mutants of *ado1Δ*, *aah1Δ*, and *adk1Δ* by crossing each of these mutants to the array strains that were chosen from those defective in signaling upstream of *PHO80* or *PHO81* (Materials and methods). The *adk1Δ* strain was defective in sporulation so we were unable to generate double mutants with this strain. We generated double mutants carrying *aah1Δ* and *ado1Δ* and measured *PHO84* expression levels in high P_i_ conditions to identify those expressing <2-fold *PHO84* more than the wild type. From these measurements, we identified 63 and 22 double mutants in the *aah1Δ* and *ado1Δ* backgrounds with elevated *PHO84* expression, respectively, with 12 of them appearing in both mutant backgrounds (S4 Table). Although there was no enriched gene ontology term in the 12 gene set, we observed that deletion of *VIP1* fully masked activation of the PHO pathway in *ado1Δ* and *aah1Δ* in high P_i_ conditions (Fig 6). Given that Vip1-synthesized IP_7_ is a signaling factor for the PHO pathway [14], this observation suggests that there may be interplay between adenine nucleotide metabolism and inositol polyphosphate synthesis.

## Discussion

In an effort to better understand signaling processes upstream of the Pho80/Pho85/Pho81 kinase complex in the PHO pathway, we used the SGA method to conduct systematic genome-wide screening. We found more than 300 mutants that are significantly defective in signaling upstream of *PHO80* or *PHO81* and investigated a subset in more detail. We found that genes acting in different metabolic pathways influence regulation of the PHO pathway. Deletion of *AAH1*, involved in adenine nucleotide metabolism, leads to derepression of the PHO pathway under high P_i_ conditions. Furthermore, Aah1 and Ado1 (another adenine metabolism gene) negatively regulate the activity of the PHO pathway in a Vip1-dependent manner, suggesting that interplay between adenine nucleotide metabolism and inositol polyphosphate metabolism may be important for regulation of the PHO pathway.

Our study provides new insight into how a decrease in ATP levels can be linked to elevation of Vip1-synthesized IP_7_ to activate the PHO pathway. This claim is supported by two observations: (1) ATP levels decrease when the PHO pathway is activated—in the wild type in low P_i_ and in *ado1Δ*, *aah1Δ*, and *aak1Δ* in high P_i_; (2) Vip1 is required for constitutive activation of the PHO pathway in the *ado1Δ* and *aah1Δ* strains. We propose that changes in ATP levels resulting from changes in P_i_ availability affect the balance between the levels of different IP_7_ isomers. Two classes of kinases, Kcs1 (IP_6_ kinase) and Vip1 (PP-IP_5_ kinase), synthesize different IP_7_ isomers from IP_6_ [30]. Furthermore, they appear to have different *K*_*m*_ values for ATP based on the values of their mammalian homologues (*K*_m_ for ATP (mM): 0.13 (Vip1) vs.1 ~ 1.4 (Kcs1)) [35]. When ATP levels are high, like the wild type in high P_i_ conditions, Kcs1-synthesized IP_7_ appears to be the dominant IP_7_ isomer as deletion of *KCS1* leads to near-complete removal of IP_7_; in *kcs1Δ*, IP_6_ levels are reduced by 20% and total IP_7_ levels are reduced by 83% compared to the wild type [36]. When ATP levels are low, as in the wild type in low P_i_ conditions (~50% decrease, Fig 4A) or in the three mutants (50 ~80% decrease, Fig 4B), we expect that Kcs1 kinase activity will be reduced but Vip1 kinase will still be active since physiological ATP concentrations in high P_i_ (1.1 ~ 1.4 mM) are near the *K*_m_ for ATP of Kcs1 [37]. These changes in Kcs1 and Vip1 kinase activities in low ATP conditions may give rise to accumulation of Vip1-synthesized IP_7_ leading to activation of the PHO pathway. A prediction of this model is that in the absence of Kcs1, Vip1-synthesized IP_7_ levels may increase due to lack of competition over IP_6_. This prediction is consistent with our observation that the *PHO84* reporter level in *kcs1Δ* is higher than that of the wild type in low P_i_ (1.33; S3 Table). To test this model, it will be necessary to carry out the following experiments: (1) to determine if IP_7_ levels in *ado1Δ*, *aah1Δ*, and *adk1Δ* mutants are comparable to those in the wild type in low P_i_; (2) to measure Vip1 and Kcs1 kinase activities as a function of ATP concentrations, detecting different IP_7_ isomer levels to determine if Vip1 is more active than Kcs1 in the low ATP regime [38].

In addition, the reduction in ATP and ADP levels in the three mutants suggests that increases in AICAR (5-aminoimidazole-4-carboxamide ribonucleotide), an intermediate of purine *de novo* synthesis, may contribute to strong *PHO84* reporter levels in the mutants. Decreases in ATP and ADP levels lead to loss of feedback inhibition of purine *de novo* synthesis [39], so it is expected that AICAR levels in these three mutants will increase. Pinson *et al*. showed that an increase in AICAR levels promotes interactions between Pho4 and Pho2, another co-transcription factor required for regulating the PHO pathway, leading to expression of the PHO regulon in a PHO pathway-independent manner [40]. Although AICAR is not likely to be acting upstream of the Pho80/Pho81/Pho85 complex [40], an increase in AICAR levels resulting from decreases in ATP and ADP levels may account for some of the increased *PHO84* reporter levels in those mutants.

When we analyzed gene ontology (GO) enrichment for the less repressed hits with DAVID [41], we found that nine genes with the GO term of “transcription elongation from RNA polymerase II promoter” are significantly enriched (p-value: 3.6*10^−4^, S6 Table). This finding suggests a possible source of cell-to-cell variability in repression of *PHO84* transcription, which may contribute to the bimodal distribution of *PHO84* expression in intermediate P_i_ conditions. The interplay of feedback loops generated by P_i_ transporter regulation creates mutually exclusive states in which cells either express *PHO84* or repress *PHO84* in intermediate P_i_ conditions [26]. Wykoff *et al*. have speculated that cell-to-cell variability leads to the bimodality in *PHO84* expression [26]. Since low copies of antisense *PHO84* RNA are expressed sporadically and are sufficient to repress *PHO84* mRNA expression within individual cells [42, 43], cells that harbor antisense *PHO84* RNA are expected to repress *PHO84* expression and the remaining cells are expected to express *PHO84*, leading to the bimodality in *PHO84* expression. As antisense *PHO84* RNA can act *in trans* [42], our *PHO84* reporter is expected to be subject to this antisense regulation. Thus, these nine genes may be involved in transcription of antisense *PHO84* RNA. This hypothesis could be evaluated by measuring antisense *PHO84* RNA in these nine mutants with single molecule RNA FISH (fluorescence *in situ* hybridization).

The observation that the PHO pathway can be activated in *vip1Δ* after a long period of time in P_i_-limited conditions demonstrates the complexity of molecular mechanisms underlying regulation of the PHO pathway. This phenotype of *vip1Δ* can be explained by two possible mechanisms: (i) alternative IP_7_ synthesis pathways where other kinases can take over the synthesis of IP_7_ in the absence of Vip1, or (ii) a novel IP_7_-independent regulation of Pho80/Pho85 kinase activity. In a future study, it may be possible to test this hypothesis by measuring IP_7_ levels in the *vip1Δ* strain over time under P_i_-limited conditions.

In conclusion, our screening results provide a resource for further studies on the molecular mechanisms by which the PHO pathway is regulated. Metabolic profiling with the mutants that we identified in this study, as a complement to genetic screening, could be useful for monitoring how different metabolic pathways—in particular, adenine nucleotide metabolism and inositol polyphosphate metabolism—respond to changes in P_i_ availability and elucidating how these pathways influence the activity of the PHO pathway. Given the complexity of the metabolic networks involved in regulation of the PHO pathway, an extensive analysis with the array strains will yield important information on the signaling network that allows budding yeast cells to respond properly to changes in environmental nutrient availability.

## Supporting information

S1 Fig The distributions of the *PHO84* reporter levels in single cells in (A) *aah1Δ* in 1mM P_i_ and in (B) *vip1Δ* in 50 μM P_i_. (TIF)

S1 Table The extracted mean *PHO84* reporter levels of the single mutants and the double mutants. (XLSX)

S2 Table The LC-MS/MS ion count data to calculate adenine nucleotide levels in Fig 4. (XLSX)

S3 Table The genome-wide screening result of the single mutants in 50 μM and 1 mM P_i_. (XLSX)

S4 Table The double mutant analysis results of *ado1Δ* and *aah1Δ*. (XLSX)

S5 Table The list of the less induced hits. (XLSX)

S6 Table The list of the less repressed hits. (XLSX)
