# Supplementary figures and images for: A systematic genetic screen for genes involved in sensing inorganic phosphate availability in Saccharomyces cerevisiae
Source: PLoS One. 2017 May 17;12(5):e0176085. doi: 10.1371/journal.pone.0176085 (PMC5435139; doi:10.1371/journal.pone.0176085)

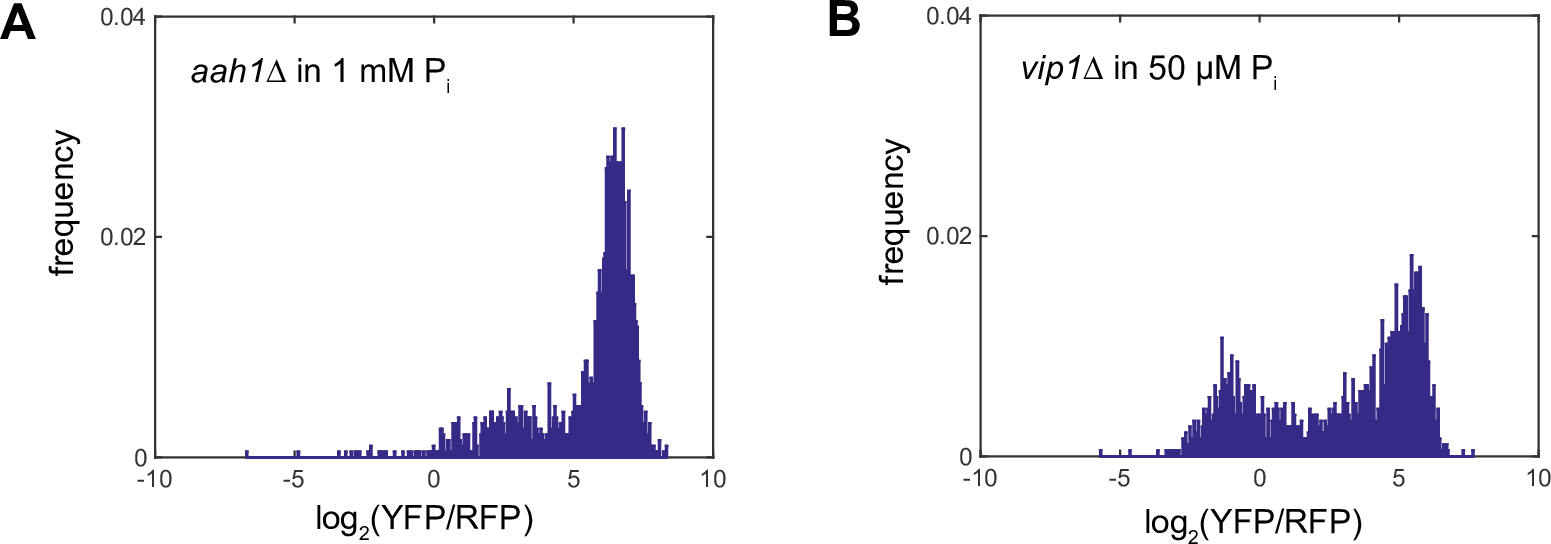

Supplement: S1 Fig — (TIF) [file pone.0176085.s001.tif]
